# MALT lymphoma associated with laryngeal amyloidosis: case report

**DOI:** 10.1016/j.bjorl.2020.12.004

**Published:** 2021-01-02

**Authors:** Leandro Castro Velasco, Laurice Barbosa Freitas, Jhessica Lima Garcia, Henrique Moura de Paula, Vitor Alves Cruz, Hugo Valter Lisboa Ramos, Claudiney Candido Costa

**Affiliations:** a Centro Estadual de Reabilitação e Readaptação Dr. Henrique Santillo (CRER), Goiânia, GO, Brazil; b Universidade Federal de Goiás (UFG), Faculdade de Medicina, Goiânia, GO, Brazil; c Universidade Federal de Goiás (UFG), Goiânia, GO, Brazil

## Introduction

Mucosa-associated lymphoid tissue (MALT) lymphoma is an extranodal marginal zone B-cell lymphoma and a subtype of non-Hodgkin lymphoma (NHL). The gastric location represents 70% of extranodal sites, and other sites include the lungs, head and neck, thyroid, skin, breast, and the larynx, among others. Its appearance in the larynx is extremely rare, corresponding to less than 1% of laryngeal neoplasms and, when present, they are more frequently found in the supraglottic region, with few reports in the subglottic and glottic regions.^1^, ^2^

It is known that MALT lymphoma has been associated with amyloid deposition in different anatomical sites, such as the gastrointestinal system, lungs, and minor salivary glands, but this location in the larynx is very rarely reported.^3^ The symptoms include dysphonia, dyspnea, dysphagia and cough. Cervical adenopathy be present. The treatment is still controversial, but most patients are treated exclusively with radiotherapy, with good results.^4^

The present is a case report of a laryngeal MALT lymphoma associated with amyloidosis.

## Case report

**Figure 1:**
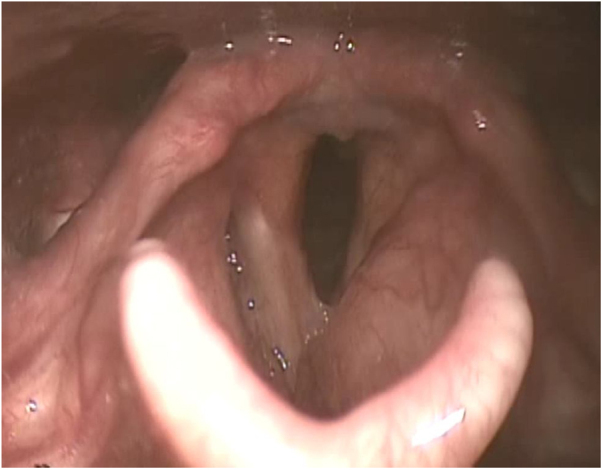
Videolaryngoscopy evaluation: infiltration of the glottic and supraglottic regions with signs of chronic laryngitis.

**Figure 2:**
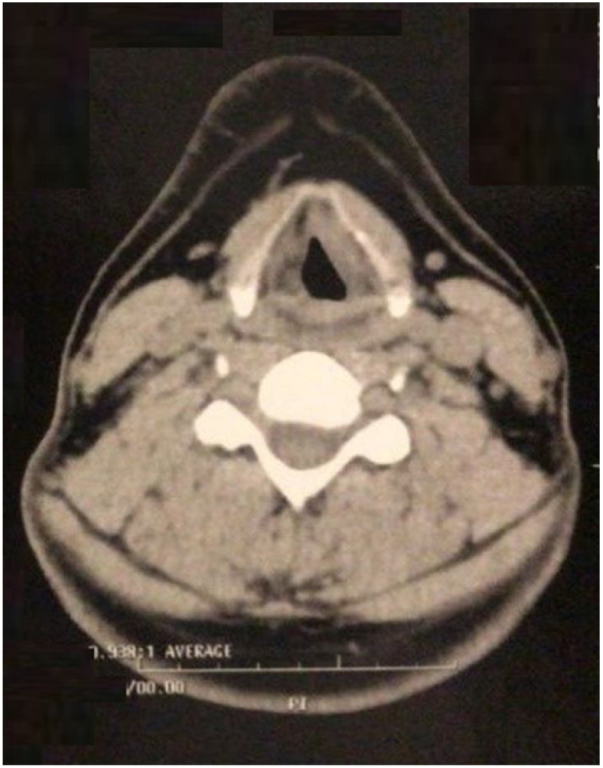
Computed tomography of the neck, axial view. Diffuse mucosal thickening of the larynx, obliteration of the paraglottic fat planes.

A male patient, 27 years old, presented with progressive dysphonia for four years. During this period, he underwent several unsuccessful drug treatments (antibiotics, systemic and inhaled corticosteroids) prescribed by different otorhinolaryngologists, and the patient refused to undergo a biopsy. After further deterioration of the vocal quality, the patient once again decided to seek care and came to our service. The videolaryngoscopy identified diffuse infiltration in the glottic and supraglottic regions, with decreased definition of the anatomical borders of the larynx and signs of chronic laryngitis (Fig. 1). There were no alterations in ganglionary chains in the cervical region. In previous examinations, diffuse mucosal thickening of the larynx and cervical tracheal segment was observed on the computed tomography of the cervical region, associated with obliteration of the paraglottic and pre-epiglottic fat planes (Fig. 2). The Laboratory tests had shown no alterations.

**Figure 3:**
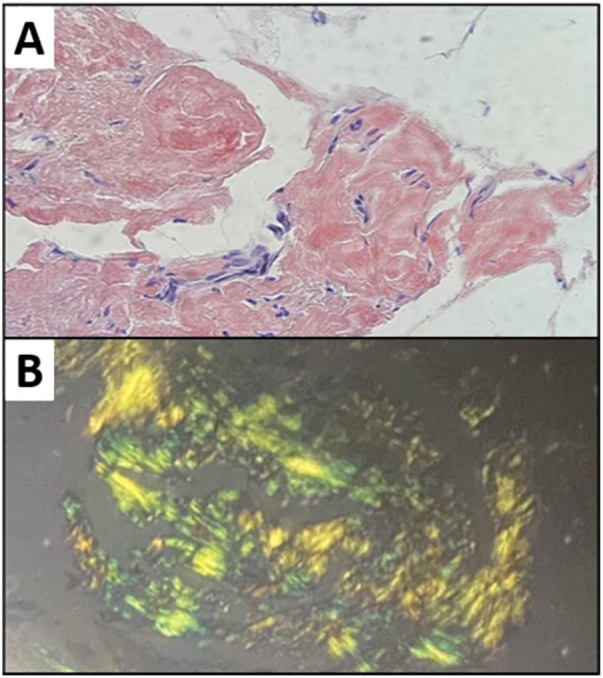
Laryngeal biopsy. (A) H&E. 40x: extensive amyloid material deposition in the chorion; (B) Congo red.

**Figure 4:**
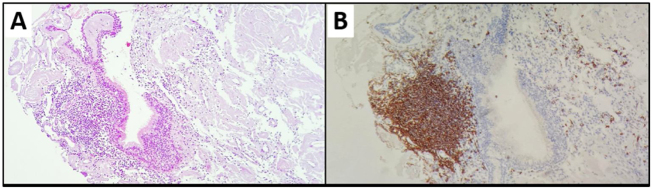
Laryngeal biopsy. (A) H&E. 10x: lymphoplasmacytic infiltrate associated with a respiratory mucosal pattern, with formation of lymphoid aggregate and peripheral plasmacytosis; (B) CD20: B-cell lymphoid aggregate.

The patient was underwent direct laryngoscopy for biopsy. The histopathological examination showed amyloidosis in a respiratory mucosal pattern (Fig. 3), associated with moderate lymphoplasmacytic infiltrate in the right vocal fold; mucosal amyloidosis was found in the left vocal fold and left ventricular border. Immunohistochemistry analysis of the right vocal fold biopsy was requested using the streptavidin technique, which disclosed several B lymphocytes (CD20-positive), several plasmocytes (CD138-positive), several Kappa plasmocytes and some Lambda plasmocytes, BCL2- and CD23-positive lymphocytes in rare cells and low proliferative index – compatible with mucosa-associated lymphoid tissue (MALT) lymphoma. Fig. 4 shows some of these alterations found in the biopsy of the right vocal fold.

**Figure 5:**
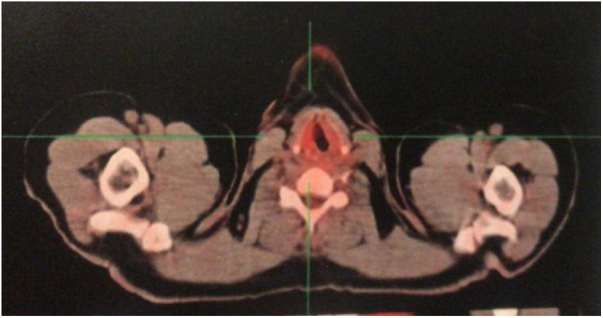
PET-CT, axial section of the cervical region. Discrete asymmetric radioconcentration.

The myelogram showed increased erythrocyte series; but no alterations of the immunophenotyping, bone marrow immunohistochemistry and histopathology. A PET-CT was performed for staging and showed slight irregularities in the right vocal cord borders, with slight asymmetric radioconcentration and grade 3 Deauville score, whereas there was no evidence of significant radioconcentration changes in the other analyzed regions (Fig. 5). The patient was underwent treatment with eighteen radiotherapy sessions and showed voice improvement.

## Discussion

Extranodal MALT lymphoma represents around 5% of all Non-Hodgkin's lymphomas (NHL), with the gastric region being the most commonly affected site. The primary MALT lymphoma of the larynx is extremely rare.^5^ According to a previous study, only approximately 90 cases have been reported in the English language literature.^6^ They occur mainly around the age of 50 years, with no difference between the genders. Symptoms can vary according to the location, including dysphonia, dysphagia, and chronic cough and it appears as a bulging smooth surface on endoscopic examination. They occur predominantly in the supraglottic region (77.3%), followed by the subglottic (18.2%) and, more rarely, in the glottic regions (less than 5%).^2^, ^5^ Regarding the pathogenesis, it is believed it has an association with autoimmune diseases or infection by *Helicobacter pylori* and *Chlamydia psittaci*, in addition to a higher incidence in immunocompromised individuals.^5^ In the present case, the patient was immunocompetent and had no diagnosis of autoimmune disease, in addition to the presence of the disease in the glottic and supraglottic regions.

Lymphoma staging is based on the Ann Arbor system, which takes into account the nodal (I to IV), extranodal (E) site involvement or both sites and their relationship with the diaphragm; in addition to separating them into stages A and B according to the presence or absence of constitutional symptoms (fever, night sweats and weight loss greater than 10% in 6 months), with patients without these symptoms being classified as type A.^4^

Due to the scarcity of studies, there is as yet no specific treatment for MALT lymphomas in the larynx, and most of them have been treated with radiotherapy alone when the disease is localized. Therapeutic options are based on research results for all extranodal MALT lymphomas. Despite the fact that radiation therapy associated with chemotherapy has a greater potential to kill tumor cells, the addition of adjuvant chemotherapy failed to show any benefit in disease-free survival and overall survival in early-stage cases.^5^ According to Rodriguez, Pérez, Ruiz, Caletrio,^2^ three cases have been described in the literature in which tumor removal and *H. pylori* eradication were the only treatments used, but none of them showed a glottic location. PET-CT has been useful for both diagnosis and staging, in addition to providing information on radiotherapy response.^2^

Even more rarely, MALT lymphoma has been associated with amyloid deposition in places such as the intestine, lungs, and minor salivary glands. Caulet et al.,^7^ reported in 1995 a case of intestinal MALT lymphoma associated with localized amyloidosis and, until that moment, no other report had been found in the literature.

Amyloidosis is a disease of unknown etiology, systemic or localized, characterized by the extracellular deposition of a protein-nature substance. Amyloid in the larynx can be identified as subepithelial extracellular deposits of acellular, amorphous and homogeneous eosinophilic material that shows apple-green birefringence under polarized light when stained with Congo red.^8^

Amyloid deposits can also be found next to primary pulmonary lymphomas and, sometimes, the amyloid is present in the lymphoma stroma, but this finding is extremely rare, occurring in less than 1% of cases. A monoclonal kappa light chain was found in both the lymphoma and amyloid deposits in a previous study and the authors strongly suggested that the amyloid was the product of a pulmonary MALT lymphoma.^9^

Thompson et al.^8^ evaluated 11 cases of laryngeal amyloidosis. Three patients were found who had developed recurrent and multifocal disease in the respiratory or gastrointestinal tract and who also had monoclonal lymphoplasmacytic infiltrate, although none of them had evident B-cell lymphoma. Thus, they suggested that some cases of laryngeal amyloidosis could be the result of a lymphoproliferative disorder originating from lymphoid tissue associated with the mucosa.^3^, ^8^

The treatment of localized laryngeal amyloidosis is primarily surgical. It is important to monitor patients in the long term for the possibility of recurrence or residual disease. Despite the low incidence, it is important to consider the association with MALT lymphoma. The performance of a clinical-radiographic and laboratory investigation is also interesting, aiming to rule out an underlying disease as well as for the adequate amyloidosis classification.^8^

The factors that predispose to the amyloid deposit in these cases need to be better elucidated, as well as the therapeutic definitions. Moreover, it is important to know these diseases to consider the presence of MALT lymphoma when amyloidosis is found in the biopsy of laryngeal tumors.

## Conclusion

The present case illustrates a case of MALT lymphoma and amyloidosis in the larynx, which contributes to the medical community as it reports two rare and associated entities in a patient who showed a favorable outcome after radiotherapy treatment.

## Conflicts of interest

The authors declare no conflicts of interest.
